# Toxicity Screening of Fungal Extracts and Metabolites, Xenobiotic Chemicals, and Indoor Dusts with In Vitro and Ex Vivo Bioassay Methods

**DOI:** 10.3390/pathogens13030217

**Published:** 2024-02-29

**Authors:** Tuomas Hintikka, Maria A. Andersson, Taina Lundell, Tamás Marik, László Kredics, Raimo Mikkola, Magnus C. Andersson, Jarek Kurnitski, Heidi Salonen

**Affiliations:** 1 Department of Microbiology, Faculty of Agriculture and Forestry, University of Helsinki, 00014 Helsinki, Finland; aino.andersson@aalto.fi (M.A.A.); taina.lundell@helsinki.fi (T.L.); 2 Department of Civil Engineering, Aalto University, 00076 Espoo, Finland; raimo.mikkola@aalto.fi (R.M.); jarek.kurnitski@aalto.fi (J.K.); heidi.salonen@aalto.fi (H.S.); 3 Department of Microbiology, Faculty of Science and Informatics, University of Szeged, H-6726 Szeged, Hungary; mariktamas88@gmail.com (T.M.); kredics@bio.u-szeged.hu (L.K.); 4 Department of Production Animal Medicine, Faculty of Veterinary Medicine, University of Helsinki, 04920 Saarentaus, Finland; magnus.andersson@helsinki.fi; 5 Department of Civil Engineering and Architecture, Tallinn University of Technology, Ehitajate Tee 5, 19086 Tallinn, Estonia; 6 International Laboratory for Air Quality and Health, Faculty of Science, School of Earth & Atmospheric Sciences, Queensland University of Technology, 2 George Street, Brisbane, QLD 4000, Australia

**Keywords:** *Basidiomycetes*, *Ascomycetes*, bioassays, boar sperm motility inhibition assay, inhibition of cell proliferation assay, fungal metabolites, biocides, detergents, indoor settled dust

## Abstract

It is controversial how useful bioassays are for identifying the in vivo toxicity of hazardous environmental exposures. In this study, fruiting bodies of forest mushrooms (n = 46), indoor mold colonies (n = 412), fungal secondary metabolites (n = 18), xenobiotic chemicals such as biocides and detergents (n = 6), and methanol extracts of indoor dusts from urban buildings (n = 26) were screened with two different bioactivity assays: boar sperm motility inhibition (BSMI) and inhibition of cell proliferation (ICP) tests. For the forest mushrooms, the toxicity testing result was positive for 100% of poisonous-classified species, 69% of non-edible-classified species, and 18% of edible-classified species. Colonies of 21 isolates of Ascomycota mold fungal species previously isolated from water-damaged buildings proved to be toxic in the tests. Out of the fungal metabolites and xenobiotic chemicals, 94% and 100% were toxic, respectively. Out of the indoor dusts from moldy-classified houses (n = 12) and from dry, mold-free houses (n = 14), 50% and 57% were toxic, respectively. The bioassay tests, however, could not differentiate the samples from indoor dusts of moldy-classified buildings from those from the mold-free buildings. Xenobiotic chemicals and indoor dusts were more toxic in the BSMI assay than in the ICP assay, whereas the opposite results were obtained with the Ascomycota mold colonies and fungal secondary metabolites. The tests recognized unknown methanol-soluble thermoresistant substances in indoor settled dusts. Toxic indoor dusts may indicate a harmful exposure, regardless of whether the toxicity is due to xenobiotic chemicals or microbial metabolites.

## 1. Introduction

Bioassays measure the cumulative effect of known and unknown bioactive substances. Bioassays do not reveal the identities of individual compounds or their origin. Testing the biological activity of chromatographic fractions and chemical identification of the active fractions are essential for effect-directed analysis (EDA) [1].

By EDA analysis, various toxic compounds have been detected, isolated, purified, and identified by the toxicological assessments of drinking water, indoor dusts, building materials, foods, and indoor microbial cultures [1,2,3,4,5,6,7]. Bioassays have been used to reveal and separate several specific toxicity mechanisms as hormone-disrupting activity and mitochondrial toxicity of xenobiotic contaminants. Specific sublethal mitochondrial toxicities of drugs, biocides, and microbial metabolites were detected in intact mammalian cells at concentrations below cytotoxic and plasma membrane-damaging concentrations [4,6,8,9,10]. EDA is instrumental for identifying hazardous environmental exposures.

There is well-documented information on the in vivo toxicity and health hazards of Finnish forest mushrooms. In cases where poisonous mushrooms are consumed, the amount of exposure and the time of onset of symptoms are known [11,12,13,14,15,16,17,18,19,20,21]. When investigating hazardous-level exposures of indoor air impurities in vivo, the causes for the reported health symptoms are difficult to detect and identify because the time between exposure and the appearance of symptoms is unknown. Mold species known to grow in wet building materials may produce mycotoxins (by definition: metabolites known to be toxic to mammals) [22,23,24]. Indoor mold species have been categorized into species able to colonize wet building materials, indicating water damage, and some of these species are known as mycotoxin-producing species [25,26,27]. Mycotoxins and toxic bacterial metabolites have been identified in contaminated indoor building materials and indoor dusts [28,29]. Xenobiotic substances as biocides, synthetic tensides in cleaning chemicals, phthalates, flame retardants, and parabens, as well as unknown substances and heavy metals, have been found in indoor dusts [30,31,32,33]. Indoor air pollution comes not only from indoor emission sources, but also from outdoor sources like air pollution, affecting the composition of particulate matter, xenobiotic chemicals, and microbes in indoor dusts [34].

Evaluating the health hazard for assessing residential safety using cellular toxicity tests in analytics of indoor dust samples is challenging because the amount and duration of exposure are unknown. Exposure to toxic molds or their toxins is expected to cause more- or less-acute symptoms. Meanwhile, exposure during pregnancy to hormone disruptors and reproductive toxins such as phthalates, triclosan, and per- and polyfluoroalkyl substances may affect the offspring in the next generation [1,35,36,37].

It has been demanded that animal experiments should be replaced by cell tests in toxicological research [8,38], but it is controversial how well cell-based bioassays replace the in vivo exposures in animal experiments. This is specifically true for the risk assessment of respiratory toxins, e.g., T-2 toxin was over 10 times more toxic when inhaled than after systemic administration [38,39]. Several mycotoxins produced by molds (e.g., ochratoxin and fumonisins) are very toxic in vivo [38,40], but there is no sensitive in vitro biotest to measure their true bioactivity. Methanol, commonly used in the extraction of toxic compounds, is a non-toxic solvent in cell tests, but its metabolic product, formic acid, is toxic to humans and monkeys [41]. Cereulide, a mitochondrial toxin produced by *Bacillus cereus,* is known to cause even fatal food poisonings in humans. It is toxic in sperm motility tests, but non-toxic to rodents [42,43]. Thus, bioassays may not always replace animal in vivo experiments to screen for harmful exposures and for risk assessment.

The suitability of two complementary bioassays was evaluated. The in vitro test was ICP (inhibition of the cell proliferation), and the ex vivo test was BSMI (boar sperm motility inhibition). We compared the results obtained from testing mushrooms, indoor molds and their metabolites, cleaning chemicals and biocides, as well as indoor dust samples. This study focused on the efficacy of the toxicity tests for the assessment of consumer safety of mushrooms and of residential safety in mold-infested and mold-free buildings.

## 2. Materials and Methods

### 2.1. Experimental Design

#### 2.1.1. Rationale for Testing Forest Mushrooms, Indoor Molds, and Indoor Dusts with the Combination of Two Complementary Bioassays

**Figure 1:**
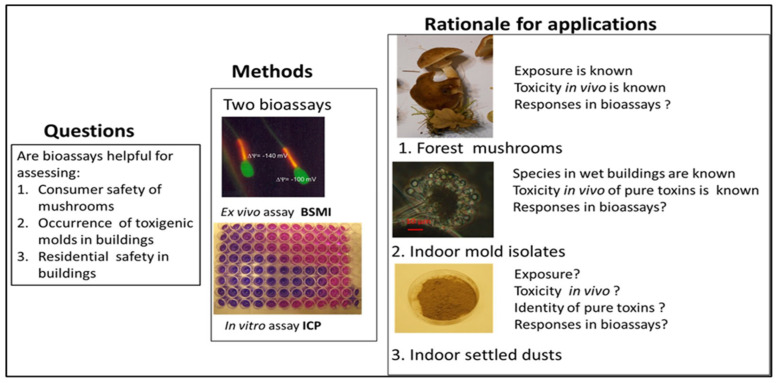
Experimental setup and rationale for the chosen applications.

The aim of the study was to evaluate the results obtained in two bioassays, considered separately and aggregated, against reported in vivo toxicities. The rationale for the experimental design and criteria for selecting the research subjects are illustrated in Figure 1 with (1) consumer safety of mushrooms, (2) toxic effects of common filamentous fungi (molds) indicating water damages in buildings and toxic effects of their metabolites, and 3) the residential safety of indoor environments of buildings by testing the toxicity of indoor settled dusts and xenobiotic chemicals (biocides and tensides used indoors for cleaning and maintenance). Information on the mycotoxins, microbial metabolites, biocides and tested chemicals is presented in Table 1. 

The rationale for choosing the three research subjects illustrated in Figure 1 were the following: (1) Mushroom poisonings described in the literature separated known poisonous species from the edible mushroom species. Toxicity testing of water extract from thawed fruiting bodies of poisonous and edible mushrooms offered the opportunity to compare the toxic responses in our bioassays to documented toxicity in vivo. (2) Toxicity testing of biomass suspensions of mold colonies, randomly isolated from problematic moldy buildings, enabled us to evaluate the ability of the two tests to recognize toxigenic indoor mold species. Identification at the species level of toxigenic isolates enabled us to recognize the toxigenic species able to grow in wet building materials and to separate different toxicity patterns exhibited by the toxins they produce. (3) Testing the intrinsic toxicity of settled indoor dusts enabled us to evaluate the ability of the tests to separate dusts from moldy water-damaged buildings from dusts from dry, mold-free buildings. The aim was to find methods for assessing the residential safety of buildings.

#### 2.1.2. Rationale for Applying Two Complementary Bioassays, Boar Spermatozoa Motility Inhibition Assay (BSMI), and Inhibition of Cell Proliferation Assay (ICP)

The rationale for using two independent and complementary bioassays for testing environmental samples was that the tests differed in sensitivity and specificity. Boar sperms are translationally and transcriptionally silent, and the BSMI assay is unsensitive to toxins affecting RNA and DNA synthesis. The BSMI assay is specific to toxins depleting mitochondrial functions and cation homeostasis [8,10]. The small volume of the sperm cell makes the BSMI assay sensitive to lethal toxins depleting plasma membrane integrity [53,54]. Cell proliferation is dependent on RNA and DNA synthesis; the ICP assay is, therefore, specific to cytostatic toxins inhibiting the synthesis of macromolecules [4,46]. In somatic cell lines, exposed as monolayers, sublethal toxins affecting mitochondria may be indicated by accelerated glycolysis [9,55,56]. Comparing the sensitivity and specificity of the two bioassays made it possible to separate the lethal or sublethal toxic responses into four different categories: (1) cytostatic toxins, (2) mitochondrial toxins, (3) toxins disturbing the cellular cation homeostasis, and (4) lethal toxins depleting plasma membrane integrity [4,53,54].

**Figure 2:**
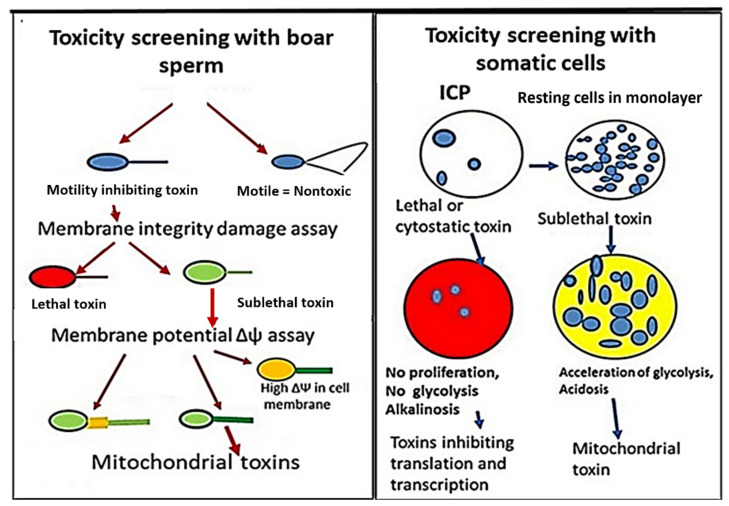
Schematic images of the two complementary bioassays, the ex vivo assay, BSMI (Boar Sperm Motility Inhibition assay), and the in vitro assay, ICP (Inhibition of Cell Proliferation assay).

Water extracts of thawed mushroom fruiting bodies and biomass dispersals of molds, as well as methanol extracts of settled indoor dusts and pure mycotoxins, and detergents and biocides used in cleaning chemicals were tested for toxicity using the two complementary bioassays, BSMI and ICP. A scheme of the two assays is shown in Figure 2. The toxicity endpoints for samples tested with the BSMI were compared to those obtained in the ICP assay, and the responses were separated into different toxicity categories.

### 2.2. Estimation of Boar Sperm Motility Inhibition—The BSMI Assay

The BSMI assay measures inability to respond to induction of motility after toxin exposure and has been described in detail previously [10,57,58]. Commercially available extended boar semen containing 2.7 × 10^7^ sperm cells mL^−1^ was obtained from Figen Ltd., Tuomikylä, Finland. In brief, after exposure for 1–3 days at 22–24 °C, motility was induced in the exposed sperm cells by shaking to provide oxygen to the sperm cells and they were warmed to 37 °C for 5 min. The induced sperm motility, i.e., progressive and rapid motility, was subjectively estimated by phase-contrast microscopy, described in Castagnoli et al. [10] and Salin et al. [57].

Phase-contrast microscopy visualizes different amplitudes of tail beating in boar sperm. In the vehicle-exposed samples, i.e., the negative control, the majority of the sperm cells exhibited rapid and progressive motility provoked by high-amplitude tail beating. These sperm cells are visible to the human eye, as an artefact consisting of sperm cells having two tails. This is easily visualized as rapid swirling motility comparable to mass activity in a microscopic frame [57]. In the negative control applied in each test, the motility of the vehicle-exposed sperm cells was estimated as the reference value of 100%. A positive (+) result in the BSMI assay is defined as a reduction in the number of rapidly motile sperms by >50% compared to that of the negative control. Briefly, the proportion of rapidly motile “two-tailed looking sperm cells” counted from 3–6 microscopic fields were <50% of the number in the vehicle-exposed sperm cells. The positive control, sperm cells immobilized by exposure to triclosan (2–4 μg mL^−1^) exhibiting motility <20% of that exhibited by the negative control, was applied in each test. When testing 100 samples, this protocol gave 99% similarity to measurements of the proportion of rapidly motile spermatozoa found by a Hamilton Thorne sperm analyzer (HTM-S, ver. 7.2; Hamilton-Thorn Research, Danvers, MA, USA). Subjectively estimated sperm motility inhibition was verified for 49 samples with a computer-based Matlab algorithm described in Castagnoli et al. [10].

Calculation of the EC_50_ concentrations of toxic substances (extracts of dust, fungal biomass and pure mycotoxins) inhibiting sperm motility in BSMI assay was performed as described [57,58,59] and Castagnoli et al. [45]. The sperm cells were exposed to the tested substances in two-fold dilutions. The EC_50_ was calculated from the equation of the straight line between EC_40–50_ and EC_80–90_: Y = −ΔY/ΔX × X + C where Y is the motility closest to 50% of the motility of the solvent control, X is the EC_50_ concentration, and C is a constant between 100% and 60%. The sperm assays were calibrated with triclosan, valinomycin, and a sample spiked with valinomycin. All tests were run in triplicate, and the average differences of the mean for three measurements were within one dilution step of two-fold dilutions. This means SD ± < 20% for measurements performed with the same batch of cells.

### 2.3. Plasma Membrane Integrity Depletion Assay and Membrane Potential Δψ Assay

Lethal and sublethal toxicity were assessed in a fluorescence microscope using 400× magnification (Nikon Eclipse E600, Nikon Corporation, Tokyo, Japan) with filters BP 450–490 nm/LP 520. The EC_50_ concentration in these microscopic assays was defined as the lowest concentration where the ratio of cells similar to those in the solvent control was less than 50%. This EC_50_ fitted between EC_90_ and EC_10_ was observed in the microscope calculating about 100–120 sperm cells from three microscopic fields. The assays were calibrated with alamethicin, a lethal necrotic toxin, and valinomycin, a sublethal mitochondrial toxin. The maximal difference between four parallel tests in each of the two methods was one dilution. Lethal toxicity in boar spermatozoa, indicated by loss of plasma membrane integrity, is described in detail in Hoornstra et al. [53,56]. Briefly, the assay is based on double staining with the viability stain propidium iodide as the dead stain and a membrane permeant cytoplasmic esterase marker Calcein-AM as the live stain (Molecular Probes Inc., Eugene, OR, USA). Lethal toxicity in PK-15 cells was performed as described in Salo et al. [52].

Sublethal mitochondrial toxicity was monitored in live sperm cells found to have intact plasma membrane integrity in the viability test with Calcein-AM and propidium iodide with the lipophilic potentiometric dye JC-1 (Molecular Probes Inc., Eugene, OR, USA). Mitochondrial toxicity, visible as depolarization of the mitochondrial membrane potential Δψm, was monitored by counting cells containing mitochondria emitting yellow (high Δψm) or green fluorescence (low Δψm), as described by Ajao et al. [9] and Mikkola et al. [60].

### 2.4. Inhibition of Cell Proliferation Assay—ICP

Cytotoxicity against somatic cells was tested with porcine tubular epithelial cells (PK-15) and feline fetus lung cells (FFL), as described earlier [4,46]. Briefly, the cells were maintained in an atmosphere of 95% air, 5% CO_2_, and 95% relative humidity at 37 °C on RPMI 1640 (complete medium) in a cell culture cabinet (Heracell 150i; Thermo Fisher Scientific, Vantaa, Finland). A 100 µL volume of detached cells, 2 × 10^5^ cells mL^−1^, was seeded onto 96-well microtiter plates. A 20 µL volume of pure chemicals, mycotoxins, and dust extracts solved in methanol or ethanol was added into 180 µL of RPMI and subjected to two-fold dilutions. The substances were tested in concentrations [dry wt mL^−1^] from below 1 ng mL^−1^ to 200 µg mL^−1^, which is the nonspecific upper limit of the assay. Ten levels of methanol or ethanol, 10% of the volume of the test cell suspension, tested in two-fold dilutions were used as negative controls. Water extracts of thawed mushroom fruiting bodies were tested in concentrations between 0.04% (*v*/*v*) and 5% (*v*/*v*). The methanol suspensions of the biomass of indoor mold colonies were tested in concentrations between 2.5% (*v*/*v*) and 5% (*v*/*v*). Toxicity endpoints were determined as inhibition of resazurin reduction, inhibition of glucose consumption, and disruption of the cell monolayer, visible in the phase-contrast microscope as described previously [46].

Sublethal mitochondrial toxicity indicated by accelerated glycolysis and depolarization of mitochondrial membranes in cells grown in monolayer was performed as described in Hoornstra et al. [56] and Ajao et al. [9]. Toxicity endpoints as EC_50_ concentrations were monitored by triplicate tests where the average difference of the mean of three measurements performed with the same batch of cells was ≤20%

### 2.5. Collection and Identification of Mushrooms

The fruiting bodies of forest mushrooms (n = 46) were collected from the Southern Finland region (Espoo, Helsinki, Mäntsälä, Lohja, Sipoo) in May–June and September–October in 2017. The fruiting bodies were collected and identified by experienced volunteer collectors and assigned to their species macroscopically [19]. Mushrooms were divided into three different groups and one sub-group. The sub-group represented edible mushrooms which must be boiled before consumption according to the instructions given by the Finnish Food Authority (Ruokavirasto, Finland).

Identification of some of the species (n = 7) was confirmed based on nucleotide sequences obtained by PCR analysis [60] applying the Thermo Scientific Phire Plant Direct PCR kit and frozen fungal fruiting body samples for analysis of the ribosomal internal transcribed spacer (ITS) region (between the nSSU/18S and nLSU/28S regions). The amplified PCR products were Sanger sequenced at BIDGEN laboratory of the Institute of Biotechnology, University of Helsinki, Finland.. The assembled sequences (GenBank Accession numbers PP152353-PP152355 and PP165371-PP165377) were analyzed with the Nucleotide BLAST service of the National Center for Biotechnology Information (NCBI) [61], and matching sequences were searched in the nucleotide collection (nr/nt) database (Appendix A). Analysis of the ITS1+ITS2 sequences revealed the identity (>98% sequence similarity) for the 7 fungal species: *Amanita fulva*, *Amanita virosa*, *Galerina marginata*, *Megacollybia platyphylla*, *Albatrellus ovinus*, *Tapinella atrotomentosa*, and *Cortinarius armillatus var. subcroceofulvus*. One species, *Tricholoma equestre*, was recognized by 95% sequence similarity. Preliminary morphological determinations were confirmed by the molecular identification.

### 2.6. Isolation of Mold Colonies

Mold colonies were isolated from settled dust collected 1.5 to 2 m above the ground level from buildings where the occupants complained about indoor air-related problems. Samples of settled indoor dusts, ca. 10 mg, were seeded on plates containing a malt extract medium (15 g malt extract from Sharlab, Barcelona, Spain and 12 g of agar from Amresco, Dallas, TX, USA, in 500 mL of H_2_O) without antibiotics or fungicides and were sealed at the site of sampling with gas-permeable adhesive tape. After three weeks of incubation at 23 ± 2 °C, the colonies on the primary isolation plates (not yet single-spored) were screened for toxicity.

### 2.7. The Toxicity Screening Assays of Molds and Mushrooms

Biomass dispersals of 412 mold colonies were screened for toxicity. The screening assays, with some minor modifications, were performed as described earlier [53,54,55,56,57]. Briefly, 20 to 30 mg biomass of the fungal colonies was dispersed in 0.2 mL methanol in a sealed glass vial and heated in a water bath of 50 °C for 10 min. Four two-fold dilutions of the obtained biomass dispersals were tested in the BSMI and ICP assays. A colony was considered toxic in the BSMI assay if <1% mL^−1^ of the biomass suspension inhibited sperm motility after 1–2 d of exposure. Cytostatic toxicity was indicated if ≤2.5% mL^−1^ of the biomass suspension inhibited cell proliferation (ICP assay) of the PK-15 cells exposed for two days [46]. In both bioassays, a nontoxic strain, *Trichoderma reesei* DSM 768, and the trilongin-producing *T. longibrachiatum* strain SzMC Thg were used as nontoxic and toxic reference strains, respectively [54]. All tests were performed in triplicate, and the average difference of the mean of three measurements performed with the same batch of cells was ≤20%, within one dilution step of two-fold dilutions.

The rapid toxicity screening assays of mushrooms were performed as follows: samples of clean, fresh fungal fruiting bodies (cap and stem parts) were identified, weighed (Mettler PM2500 Deltarange, Mettler Toledo International Inc., Columbus, OH, USA), and frozen (−20 °C). After thawing, ca. 2 mL of the liquid biomass dispersals secreted from the broken cells was pipetted into a glass vial (VWR International LLC, Radnor, PA, USA), weighed (Sartorius BP221D, Sartorius AG, Göttingen, Germany), pasteurized (heating in a bath at 70 °C, 5 min), and used for toxicity screening. The water extract of thawed fruiting bodies was considered toxic in the BSMI assay if <1% mL^−1^ of the water extract of thawed fruiting bodies inhibited sperm motility after 1 d of exposure. Cytostatic toxicity was indicated if ≤5% mL^−1^ of the water extracts inhibited cell proliferation (ICP assay) of the PK-15 cells exposed for 2 d. The positive control was a 10 mg mL^−1^ aqueous solution of triclosan, and tested concentrations were two-fold dilutions, 1–10 μg mL^−1^ in the BSMI assay and 4–16 μg mL^−1^ in the ICP assay. The negative control was the commercial white button mushroom, *Agaricus bisporus* (Mykora Oy, Kiukainen).

### 2.8. Toxicity Testing of Pure Substances and Mycotoxins with the BSMI and ICP Assays

The test solutions of the chemicals were made in water, methanol, or ethanol depending on their solubility properties. Toxicity of pure substances and mycotoxins was monitored as toxicity endpoints for EC_50_ concentrations as described in Mikkola et al. [58] and Castagnoli et al. [10]. Basically, in the BSMI assays, EC_50_ values < 25 µg mL^−1^ were considered as toxic, while EC_50_ values > 50 µg mL^−1^ were recorded as nontoxic. In the ICP assay, EC_50_ values < 50 µg mL^−1^ were recorded as toxic, while EC_50_ values > 100 µg mL^−1^ were recorded as nontoxic.

In somatic cell lines, a positive readout for sublethal mitochondrial toxicity was indicated by EC_50_ concentrations for accelerated glycolysis below one-tenth of the EC_50_ for cell death, measured as inhibition of glycolysis. In boar spermatozoa, a positive readout (+) for mitochondrial toxicity was indicated if EC_50_ concentrations for mitochondrial depolarization were below one-fourth of the EC_50_ concentration indicating a loss of plasma membrane integrity and lethal necrotic cell death.

### 2.9. Toxicity Testing of Extracts of Settled Indoor Dusts

Settled dusts collected >1.5 m above floor level were sampled from water-damaged problematic houses (n = 12) and dry non-problematic houses (n = 14). Settled dusts were extracted by methanol, and the EC_50_ concentrations for the methanol-soluble dry substances were calculated as described earlier [4]. In the BSMI assay, EC_50_ values < 25 µg mL^−1^ were interpreted as a positive toxic response and EC_50_ values > 50 µg mL^−1^ as a negative response. In the ICP assay, EC_50_ < 50 µg mL^−1^ was interpreted as a positive toxic response.

### 2.10. Identification of the Toxigenic Molds

Colonies screened as toxic were pure-cultured on malt extract agar. The pure cultures were divided into 22 morphotypes, shown in Supplementary Figure S1. One strain (n = 22) of each morphotype was identified at the species or genus level by analysis of marker gene sequences. The methods used and the accession numbers of the deposited sequences of the marker genes (ITS, *rpb2*, and CaM) are documented in [6,24,45,51,52,59].

### 2.11. Reagents and Supplies

The continuous cell lines used in the ICP assay, PK-15 (porcine kidney cells) and FFL cell lines, were provided by Finnish Food Authority (Helsinki, Finland). The commercial extended boar semen was obtained from Figen Ltd. (Tuomikylä, Finland). Commercially available tensides, biocides, mycotoxins, and fungal metabolites are presented in Table 1. The information listed is from the safety data sheets provided by the producers. The other chemicals were of analytical grade and purchased from local suppliers.

## 3. Results

### 3.1. Toxicity of Forest Mushrooms

The responses of poisonous mushrooms (n = 8), non-edible mushrooms (n = 16), and edible mushrooms (n = 22) were compared in two complementary toxicity tests. In vivo toxicities of 46 mushroom species were divided into three different categories, as shown in Table 2. The specific toxicities of poisonous mushrooms and non-edible mushroom species were higher than that of edible mushrooms. The combined result of two complementary toxicity tests gave a positive response for 8/8 (100%) of the poisonous mushroom species, 11/16 (69%) of the non-edible species, and 4/22 (18%) of the edible mushroom species.

For a combined group of poisonous and non-edible mushrooms (n = 24), tests gave a positive response for 19/24 (79%), and when evaluated separately, both the BSMI and the ICP test gave a positive response for 14/24 and 10/23 (58% and 43%), respectively, for this group. For the edible mushroom group, the response was positive for 4/22 (18%) species when the tests were evaluated together, and when evaluated separately, BSMI and ICP tests gave a positive response for 2/22 (9%) and 2/22 (9%), respectively, for this group.

By combining the toxicity tests, sensitivity improved, but specificity suffered. The specific toxicity in these tests did not distinguish poisonous mushrooms from edible ones with full certainty; however, the difference of 100% versus 18% is statistically significant (χ2 test *p* < 0.01). Three out of the eight tested poisonous mushrooms (*Amanita virosa*, *Inocybe geophylla*, and *Lactarius helvus*), two of the sixteen non-edible mushrooms (*Megacollybia platyphylla*—formerly *Clitocybula platyphylla*—and *Tapinella atrotomentosa*), and none of the twenty-two edible mushrooms were toxic in both the BSMI and ICP assays. Three out of the poisonous mushrooms, mentioned above, five out of the sixteen non-edible mushrooms (*Cortinarius armillatus*, *Clitocybe fragrans, Clitocybe vibecinna, Hygrocybe conica*, and *Hygrophorus erubescens*), and two out of the twenty-two edible mushrooms (*Clitocybe odora*, and *Leccinum versipelle*) were toxic in the BSMI assay only. One of the poisonous mushrooms (*Galerina marginata*), four of the sixteen non-edible mushrooms (*Leotia lubrica*, *Phaeolepiota aurea*, *Pholiota lenta*, and *Pholiota squarrosa*), and two of the twenty-two edible mushrooms (*Albatrellus ovinus* and *Leccinum scabrum*,) were toxic in the ICP assay only. Among the well-known edible mushrooms, *Leccinum versipelle*, *Leccinum scabrum*, and *Albatrellus ovinus* were toxic in the cell tests. Out of 24 known poisonous and non-edible mushrooms, *Calocera viscosa*, *Cortinarius collinitus*, *C. crassus*, *C. traganus*, and *Tricholoma equestre* tested negative in both toxicity assays.

**Figure 3:**
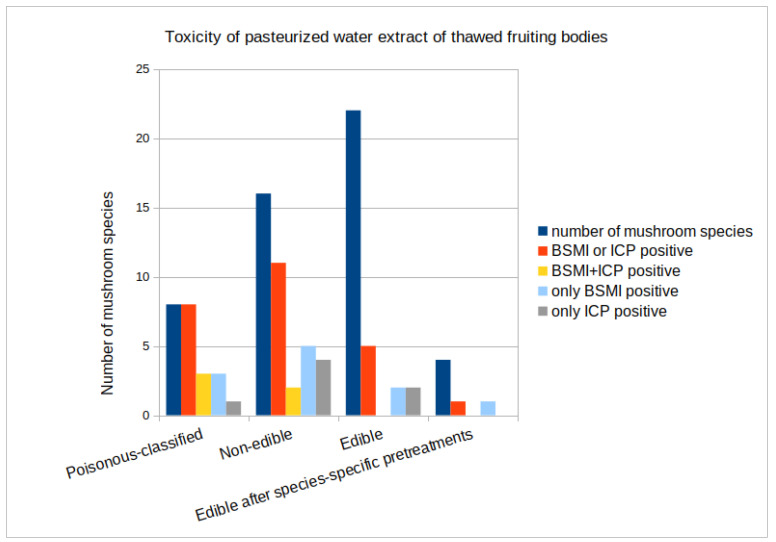
The four toxicity profiles obtained for pasteurized water extracts from thawed fruiting bodies of poisonous, non-edible, and edible mushroom species (n = 46) in the BSMI (boar sperm motility inhibition) and ICP (inhibition of cell proliferation) assays. The red bar, BSMI- or ICP-positive, indicates the number of samples positive in either of the two assays. The green bar, only BSMI-positive, indicates number of samples tested positive specifically in BSMI assay but negative in ICP assay. The gray bar, only ICP-positive, indicates samples tested positive specifically in the ICP assay but negative in the BSMI assay. The yellow bar, BSMI+ICP-positive, indicates the number of samples positive in both assays.

The 46 tested mushrooms exhibited four different toxicity profiles: 39% were toxic in either the BSMI or ICP assay, 11% of the 46 tested mushrooms were toxic in both assays, 15% were toxic in the ICP assay only, and 22% were toxic in the BSMI assay only. Out of the tested mushrooms, 12/46 (26%) were toxic in the ICP assay, while 16/46 (35%) were toxic in the BSMI assay. A summary of the toxic responses of the pasteurized water extracts from the 46 tested mushroom species is presented in Figure 3. The figure illustrates the four toxicity profiles and their occurrence among the poisonous, non-edible, and edible mushrooms. The red bars indicate the sensitivity of the aggregated results of the two assays, whereas the blue bars express the specificity of each assay evaluated separately. The yellow bars show the number of samples giving positive readout in both of the assays.

### 3.2. Toxicity of Indoor Mold Isolates

We screened biomass suspensions of 412 single colonies of ascomycetous molds isolated from indoor samples from 46 damp, moldy houses. Of these, 265 colonies (64%) gave a toxic response in the BSM and ICP assays. Representants of the toxic colonies were pure-cultured and divided into 22 morphotypes based on their morphology, toxic responses, and pathogenic potential (ability to grow at neutral pH at 37 °C). Supplementary Figure S1 shows the morphology of the pure cultures.

The results in Table 3 show the selected strains of each morphotype identified at the species level, the toxic responses of their biomass suspensions, and the toxic compounds identified. The results also show that the two toxicity assays revealed three different toxic responses. The majority of the strains, 11/21 (52%), were toxic in the ICP assay only; 2/21 (10%) were toxic in the BSMI assay only; and 8/21 (38%) were toxic in both tests. Out of the tested strains, 19/21 (90%) were toxic in the ICP assay, while 10/21 (48%) were toxic in the BSMI assay.

### 3.3. Toxicity of known Mycotoxins, Fungal Metabolites, and Xenobiotic Chemicals

Specific toxicities expressed as 50% effective concentration values (EC_50_) of the purified toxins, pure toxins, and xenobiotic chemicals are shown in Table 4. For the microbial metabolites, EC_50_ < 5 µg mL^−1^ and <10 µg mL^−1^ were considered as toxic in the BSMI and ICP assays, respectively. For the xenobiotic chemicals, EC_50_ values < 10 µg mL^−1^ and <20 µg mL^−1^ were considered as toxic in the BSMI assay and in the ICP assay, respectively (Table 3). For mitochondrial toxicity, a positive readout was interpreted if acceleration of glycolysis was observed at <10 μg mL^−1^ and at 10-times-lower exposure concentrations than the lethal toxicity.

The results showed that the 18 microbial metabolites exhibited three different toxicity profiles representing three different responses in the two bioassays. Out of the tested metabolites, 7/18 (39%) were toxic in both assays, and 1/18 (6%) of the metabolites was not toxic in either of them. Out of the tested metabolites, 5/18 (28%) were >100 times more toxic in the ICP assay and classified as toxic in the ICP assay only, while 4/18 (22%) were >10 times more toxic in the BSMI assay and classified as toxic in the BSMI assay only. Out of the 18 metabolites, 13 (72%) were toxic in the ICP assay and 12 (67%) were toxic in the BSMI assay.

Known mitochondrial toxins were characterized by low EC_50_ concentrations in the BSMI assay compared to the EC_50_ values in the ICP assay. Toxic substances inhibiting translation and/or protein synthesis (i.e., sterigmatocystin, satratoxin, and T2-toxin) were characterized by low EC_50_ values in the ICP assay. Toxins that form ion channels in cell membranes (i.e., alamethicin, trichorzianines, and trilongins) and toxins that inhibit glucose transport (chaetoglobosins) were toxic in both the BSMI and ICP tests. These positive responses in both tests were also exhibited by toxins with unknown effects. The results in Table 3 and Table 4 indicate that the toxicity responses of the pure toxin and the producer strains corresponded to each other, suggesting that the purified toxin likely was responsible for the major toxic response in the bioassay.

The toxic responses of the xenobiotic chemicals are shown in Table 4. The biocide triclosan and the detergents Genapol X 080, SDS, and Triton X-100 exhibited >10-times-lower EC_50_ concentrations in the BSMI assay compared to the EC_50_ concentrations in the ICP assay. However, the biocide didecyldimethylammonium chloride (DDAC) was highly toxic in both assays. Mitochondrial toxicity was recorded for triclosan, DDAC, and for Genapol X 080 after testing with somatic cells. None of the xenobiotic chemicals were toxic in the ICP assay only. The xenobiotic chemicals exhibited two different toxicity profiles: most of the chemicals, five of the six (83%), were 10 times more toxic in the BSMI assay than in the ICP assay only, while one of the six (17%) chemicals was equally toxic in both assays, whereas none of the chemicals were toxic in the ICP assay only. Out of the tested chemicals, all were toxic in the BSMI assay, and two of the six were toxic in the ICP assay.

No information available about in vivo toxicity, as the substance is not commercially available or not mentioned on the safety sheets of Sigma-Aldrich.

The results in Table 2, Table 3 and Table 4 show that our toxicity tests identified three different toxicity profiles (types of toxic responses) in the mushrooms, indoor molds, pure mycotoxins, and microbial metabolites, whereas the xenobiotic chemicals exhibited two toxicity profiles. Most of the mushrooms and molds were toxic in the ICP assay, whereas the xenobiotic chemicals were non-toxic. Interestingly, the biocides and detergents were toxic in the BSMI assay, and the biocides and Genapol X 080 seemed to exhibit mitochondrial toxicity. The results in the three tables show that in the toxicity tests, toxin-producing basidiomycetous and ascomycetous isolates, selected pure mycotoxins produced by indoor ascomycetous isolates, and, in addition, xenobiotic chemicals and detergents were detected by the assays used. However, a common poisonous mycotoxin, ochratoxin A, was not detected in our assays.

### 3.4. Toxicity of Settled Indoor Dusts

Next, we tested the toxicity of methanol extracts of indoor settled dusts. We compared the responses of dust extracts from dry houses (n = 14) and damp, moldy houses (n = 12) using the BSMI and ICP tests. The results in Table 5 show that 6/12 (50%) of the samples collected from the moldy houses were toxic in either the BSMI or ICP test or both. Of the samples from dry houses, 8/14 (57%) were toxic. Our toxicity tests did not distinguish between dust isolated from moldy houses and dust from dry houses. However, our tests separated extracts from good quality hay from extracts prepared from feed and bedding material spoiled by microbes. The 26 extracts prepared from settled indoor dusts exhibited two different toxicity profiles: 7/26 (27%) of the dusts were toxic in the BSMI assay only, and 6/26 (23%) were toxic in both assays, respectively. None of the dusts were toxic in the ICP assay only. When the dusts from moldy buildings and dusts from dry, mold-free buildings were considered separately, 3/12 (25%) of the dusts from moldy buildings were toxic in the BSMI assay only, and 2/12 (17%) were toxic in both assays, while 4/14 (29%) of the dusts from the dry buildings were toxic in the BSMI assay only, and 4/14 (29%) were toxic in both assays. Out of the twenty-six tested dust extracts, thirteen (50%) were toxic in the BSMI assay and six (23%) were toxic in the ICP assay.

The results also show that the dusts that inhibited sperm motility in the BSMI sperm test were toxic to mitochondria in somatic cells. Three of the dusts from moldy houses and six of the samples from dry, mold-free houses tested positive for mitochondrial toxicity. The biocides tested, DDAC, triclosan, as well as the detergents Genapol X 080, Triton X-100, and the positive controls (dusts emitted from spoiled feed and bedding connected to sickness of exposed animals and hay dust spiked with the known mitochondrial toxin valinomycin), all gave positive (+) toxic responses in the tests. Dusts from good quality hay fed to horses healthy at the moment of dust sampling were not toxic in any of the tests.

### 3.5. Summary of Toxicity Responses

The results of Table 2, Table 3, Table 4 and Table 5 are summarized in Table 6, showing that the mushrooms, indoor mold colonies, and fungal metabolites exhibited three different toxicity profiles. The responses included toxicity in the BSMI assay only, toxicity in the ICP assay only, and toxicity in both assays. The proportion of total positive responses in the ICP assay was higher than or almost equal to that recorded in the BSMI assay. The xenobiotic chemicals and the settled dust exhibited two different toxicity profiles: toxicity in the BSMI assay only and toxicity in both assays. No responses that were positive in the ICP assay only were recorded for the settled dusts. Also, the proportion of total positive responses in the BSMI assay was higher than that recorded in the ICP assay.

## 4. Discussion

In this study, results obtained in the ex vivo assay, BSMI, and in the in vitro assay, ICP, were evaluated against hazardous toxic exposures documented in vivo. To our knowledge, this approach for evaluating toxicity measured in these bioassays against toxicity recorded in vivo has not previously been applied or reported.

Mushroom poisons causing human poisonings are well-described in the literature [11,12,13,14,15,16,17,18,19,20]. Water extracts of fruiting bodies of basidiomycete and ascomycete species that are known to be toxic to humans offered our study the opportunity to compare the in vivo toxicity of forest mushrooms with the toxicity responses of in vitro and ex vivo cell tests. In the BSMI and ICP cell tests, the specific toxicity of known poisonous mushroom species was higher (100%) than that of edible mushroom species (18%), but the tests did not distinguish poisonous mushroom species from edible species completely and with an absolute certainty. *Leccinum versipelle*, classified as an edible mushroom, has been reported to cause symptoms of intoxication when consumed without proper heat preparation [63], while *Albatrellus ovinus* is known to be toxic to human amnion U-cells [64]. There are three hypothetical reasons for the positive cell test results of four edible mushrooms:

(A) Pasteurization is a milder heat treatment than the heat treatment of conventional mushroom food preparation with many species. Sporadic cases with poisoning symptoms connected to the consumption of these species have been reported in Finland [63]. The symptoms may have been caused by an unknown, moderately heat-labile toxin.

(B) Toxins are converted by human metabolism into less harmful compounds.

(C) The mushrooms were contaminated by toxigenic molds.

The two complementary ex vivo and in vitro bioassays, evaluated separately and aggregated, detected novel toxic responses in water extracts of thawed fruiting bodies from mushrooms connected to documented cases of poisonings in vivo and from mushrooms considered as non-edible. Regarding the toxicity of the wild forest mushrooms, there has been no single test that reliably distinguishes all poisonous mushrooms from non-toxic mushrooms (“no rule is the only rule”) [65]. The mushroom part of this study concentrated mostly on water-soluble compounds; no extra solvents to extract the different chemical compounds of the fruiting body were used. Some fungal toxins, e.g., gyromitrin in *Gyromitra esculenta*, are water-soluble and highly volatile [66]; on the contrary, orellanine, found in some *Cortinarius* species, is insoluble in water [67]. The solvent dependency while extracting different chemical compounds from a mushroom fruiting body has been studied [68] and could also be addressed in future studies. After the fruiting body is picked up from the forest, its dry-matter content starts to vary according to different environmental variables (i.e., relative humidity and temperature); also, the form and age of the mushroom fruiting body affect the dry-matter content [69]. A positive result is an indicator of the need to continue a more chemistry-oriented approach with the different extraction steps and full-scale chemical analyses. To our knowledge, toxins inhibiting boar sperm motility, possibly indicating mitochondrial toxicity, have not been detected from basidiomycetous mushrooms.

The rationale for using the two independent and complementary bioassays, BSMI and ICP, may seem confusing at the first sight. However, the novelty and advantages are provided by the comparison of the different sensitivities of the assays. The different sensitivities indicated by the toxicity endpoints, 50% effective concentrations (EC_50_) in the BSMI and ICP assays, made it possible to separate the tested samples into different toxicity profiles (Figure 2 and Figure 3). The different toxicity endpoints obtained in the assays also reflected the different biological targets of the substances included in the tested samples.

The results from different applications, mold colonies, fungal metabolites, indoor dusts, and xenobiotics included novel information useful for assessing safe and hazardous environmental exposures. The difference in the toxicity profiles obtained for indoor fungi and xenobiotics potentially contaminating indoor dusts revealed information about the emission source of the toxic substances. This is a key point for monitoring the residential safety of buildings. Our tests revealed new bioactive agents produced by indoor fungi and new biological activities of pure fungal metabolites and xenobiotic chemicals used indoors (Table 2, Table 3 and Table 4). However, our tests did not distinguish the intrinsic toxicity of the settled dusts from mold-infested and mold-free buildings (Table 5). These tests did not identify the dry, mold-free buildings as safe for their inhabitants.

Our bioassays recognized toxigenic colonies representing toxigenic indicator species for water damage among indoor molds randomly isolated from water-damaged buildings [23,24,26]. Interestingly, the assays also detected new toxigenic species, *Acremonium exuviarum* and *Acrostalagmus luteoalbus*, not previously known to colonize indoor building materials [6,62]. The 265 colonies screened as toxic out of the 412 tested colonies isolated from water-damaged buildings represented 21 species of filamentous ascomycete fungi. Many of these genera (*Aspergillus, Chaetomium, Paecilomyces, Penicillium, Stachybotrys*, and *Trichoderma*) are known to produce toxic metabolites and mycotoxins and to colonize water-damaged buildings [23,26,27,28,29]. Representants of these species are also connected to building-related health complaints and are known as opportunistic pathogens [24]. Our tests thus recognized the most common mycotoxin producers known to grow in wet building materials in Finland [24,26], but we do not know the species composition and potential mycotoxin production of the colonies screened as negative in our toxicity assays. However, none of the toxigenic strains isolated from Finnish buildings produced mycotoxins specifically affecting mitochondria, like acrebol, enniatin, moniliformin, emodin, or viriditoxin (Table 3 and Table 4). Our positive test results were confirmed, but the possible false-negative results remained in the dark.

As shown in Table 5 and Table 6, xenobiotic chemicals and indoor settled dust shared more pronounced toxicities measured in the BSMI assay compared to the toxicity in the ICP assay. Fungal biomass dispersals and identified metabolites of Finnish indoor fungal metabolites (Table 3 and Table 4) exhibited stronger toxicity in the ICP assay. Boar spermatozoa motility was inhibited by phthalates, heavy metals, biocides, and detergents [70,71]. Our tests did not separate the 12 dusts from the mold-infested buildings from the 14 dusts from the mold-free buildings. The intrinsic toxicity of these 26 dusts tested did not separate the mold-infested buildings from the mold-free buildings. In fact, three of the “moldy dusts” and six of the “mold-free dusts” contained hydrophobic and at least moderately heat-resistant toxic substances affecting mitochondria in the bioassays with mammalian cells. The dusts contained mitochondrial toxins regardless of whether the samples came from moldy “contaminated” or mold-free “healthy” buildings. If mycotoxins had been the only or even the major toxins present in the house dusts, it could have been expected that the tests would separate moldy house dusts from mold-free house dusts. Bacteria such as *Streptomyces* and *Bacillus cereus* isolated from indoor dusts and building materials produce mitochondrial toxins [5]. Since this was not the case, the house dusts very likely contained additional anthropogenic mitochondrial toxins visible in our assays but of non-mold origin.

The intrinsic toxicity of indoor dust in buildings may indicate harmful exposure, but for these particular 26 dust samples, the toxicity results were impossible to relate to exposure and symptoms. This agrees with previously reported results [72,73] showing dust samples from moist buildings not differing in intrinsic toxicity from dusts from dry buildings. However, the results of other studies showed that intrinsic toxicity of indoor dust increased the risk of the exposed persons to develop building-related symptoms [49,74]. Indoor settled dusts are very heterogenous in the composition of substances and intrinsic toxicity. Different toxic substances may be soluble in different solvents; no standard extraction protocol would be optimal for all indoor dust samples. Also, the sensitivity of the exposed persons to develop symptoms may differ. The settled dusts and the exposed persons fit a classic Wittgenstein’s “Familienähnlichkeit” situation: a set of common traits or occurrences of which all have some, and none have all [75]. An association between the toxicities of certain sets of dusts and certain acute symptoms recorded in exposed persons may be found, but the association may be absent for other sets of dusts and other exposed persons. This does not necessarily mean that either survey gave wrong results. Occupants becoming ill with building-related symptoms in every situation is an outcome that can never be exactly replicated in a scientific experiment [75].

The health hazard of dusts depends on the concentration of dust particles in the air. In urban environments, the concentration of airborne dust is usually low, <10 mg m^−3^. However, the airborne concentrations of dusts and impurities are not static conditions; they are highly dependent on activities indoors and air flows caused by ventilation [76,77]. Cleaning, moving from one apartment to another, and searching for goods may occasionally rise the concentrations of airborne dusts and increase the exposure [78]. Based on the results of our study, without chemical analytics, it is impossible to know whether the toxicity of the “moldy house dust” was due to microbial metabolic products or chemicals produced by humans.

Mitochondrially toxic, manmade chemicals such as reproductively toxic biocides, triclosan and quaternary ammonium disinfectants (DDAC, [78,79]), heavy metals, flame retardants, triclosan, parabens, and phthalates, have been found in indoor dust [33,79,80,81,82,83]. Today, more than 1000 chemicals are known to be in use, the biological effects of which have not been tested [36].

Persistent, so-called environmentally toxic chemicals (e.g., triclosan and DDAC) enter waterways and the atmosphere, may enrich in food chains, and, in the long term, harm the reproduction and health of humans and other organisms [1,30,84,85]. The time of exposure to these substances during fetal development can determine the appearance of symptoms and level of harm to health [36,86,87].

Indoor dust and air may represent an exposure source for environmental mitochondrial toxins. Exposure to mitochondrial toxins, specifically, to persistent anthropogenic mitochondrial toxins, is recognized as a potential hazard affecting both indoor and outdoor ecosystems [88,89,90,91,92].

Toxins harmful in vivo such as ochratoxin and methanol represented false-negative responses in our bioassays compared to known in vivo responses. Detergents such as Tween 80 gave a weak positive response in the BSMI assay, but no respiratory toxicity in vivo was reported. The harmful respiratory toxicity recorded for tensides is mainly linked to their ability to increase permeability of the plasma membrane, potentiating the effects of additional coexisting toxic substances [93]. This study demonstrates the need of several analytical techniques, biological and chemical, used in parallel when exposures hazardous to human health are monitored.

No single bioassay can distinguish a microbial toxin from a toxic chemical, whether the target cell is a bacterium, a mammalian cell, a human cell, or a multicellular organism. The specific toxicity of the dust can still indicate a possible harmful exposure. The toxic compounds in the dust should be identified by chemical methods and thus attempt to identify the origin of the poison and the emission source so that harmful exposures can be prevented. The heterogeneity of indoor dusts and the small amounts and wide range of toxic substances present challenges for extraction methods.

## 5. Conclusions

In this study, the number of samples from mushrooms, mold colonies, xenobiotics, and indoor dusts screened for intrinsic toxicity was moderate. Still, in our opinion, the study illustrates the benefits of using two different but complementary bioassays for tracking toxicity in environmental samples.

The toxicity of poisonous forest mushrooms is an internationally accepted fact based on peer-reviewed science. The use of two complementary tests and the pasteurized water extracts of thawed fruiting bodies as a solvent was able to differentiate the poisonous Finnish forest mushrooms from the edible ones in this study. The use of different solvents to extract the different chemical compounds of the fruiting body could provide more information about the biologically active compounds of the mushroom fruiting body.

By comparing the differences in the toxicity endpoints of the two bioassays, four different positive responses were obtained, dividing the samples into four toxicity profiles:

(1) Samples inhibiting either sperm motility or cell proliferation (BSMI- or ICP-positive);

(2) Samples that inhibited sperm motility only (only BSMI-positive);

(3) Samples that inhibited cell proliferation only (only ICP-positive);

(4) Samples that inhibited both sperm motility and cell proliferation (BSMI+ICP-positive)

Samples that were negative in both tests represented a fifth toxicity profile.

A comparison of the toxicity profiles obtained with the two assays revealed possible cellular and organellar targets of the toxic substances: the mitochondria, the plasma membrane, ion homeostasis, and the synthesis of macromolecules. The bioassays recognized the eight poisonous mushrooms and recognized toxigenic indoor mold species known as indicator species for water damage. The assays recognized tensides and biocides as well as fungal metabolites known as toxic in vivo to mammals and fishes.

The results of this study indicate that 10 out of the 26 settled indoor dust samples contained lipophilic thermoresistant mitochondriotoxic substances. The tests did not separate the settled dusts from mold-infested and mold-free buildings. Based on the toxicity profiles exhibited by the xenobiotic chemicals and the settled indoor dusts and their strong toxic responses in the BSMI assay, we felt tempted to conclude that the toxic substances in the settled dusts were very likely xenobiotic chemicals of human origin. If the substances were regarded as xenobiotic, bioaccumulation in the environment may cumulate the hazardous effects in outdoor ecosystems.

## Figures and Tables

**Table 1:** Commercially available detergent biocides, fungal metabolites, and chemicals used in the study.

	Detergent				
	Chemical Name	Type	Toxicity In Vivo LD_50_		Reference
			**Fish** **mg L^−1^**	**Rat p.o.** **mg kg^−1^**	
Genapol-X-080 ^†^	Polyethyleneglycol monalkyl ether	nonionic	5	>2000	[44]
Triton X-100 ^†^	Polyethyleneglycol-p-is-octylphenyl ether	nonionic	5	500	[44]
TWEEN 80 ^†^	Polyethylene glycol sorbitan mono oleate	nonionic	Not known	>25,000	[44]
SDS ^†^	Sodium dodecyl sulfate	anionic	30	1288	[44]
	Biocides				
	Chemical name	Biological activity	Toxicity in vivo LD_50_		
			Fish mg L^−1^	Rat p.o. mg kg^−1^	
Triclosan ^†^	Triclorooxy-phenylether	Mitochondrial toxin	0.5	3700	[9,44]
DDAC *	Didecyl- dimethyl- ammonium- chloride	Mitochondrial toxin	0.5	238	[44]
	Fungal metabolites				
	Chemical name	Biological activity	Toxicity in vivo LD_50_ Rat p.o. mg kg^−1^		Ref.
Alamethicin ^†^	Peptaibol	Cation channel-forming ionophore	80		[45]
Citrinin ^†^	Polyketide	Not known	105		
Ophiobolin A **	Sesterterpene	Not known	238		[46]
Chaetoglobosin A ^†^	Cytochalasin	Inhibitor of glucose transport	>400		[47]
Emodin ^†^	Antraquinone	Mitochondrial toxin	1000		[48]
Enniatin B ^†^	Depsipeptide	K^+^ channel-forming inophore	350		[49]
Moniliformin ^†^	Dione	Mitochondrial toxin	50		[50]
Ochratoxin A ^†^	Isocoumarin	Not known	20		[51]
Sterigmatocystin ^†^	Polyketide	Inhibitor of translation	120		[52]
T-toxin ^†^	Trichothecene	Inhibitor of translation	2.7		[4]
Roridin ^†^	Trichothecene	Inhibitor of translation	55		[4]
Verrucarin ^†^	Trichothecene	Inhibitor of translation	5.1		[4]
Valinomycin ^†^	Depsipeptide	Mitochondrial toxin	4		[10]

* Merck, Darmstadt, Germany. ^†^ Sigma-Aldrich, St. Louis, MO, USA. ** Purified as described in [46].

**Table 2:** Toxicity of pasteurized water extract from thawed fruiting bodies of poisonous, non-edible, and edible mushroom species (n = 46) in two complementary bioassays. Documented toxicity of mushroom species is marked as toxicity in vivo. The ex vivo BSMI assays measure boar sperm motility inhibition. The in vitro ICP assay measures inhibition of cell proliferation of the somatic continuous porcine kidney cell line PK-15. A positive response (+) in the assays was defined as when 1% of water extract of thawed fruiting bodies inhibited the boar sperm motility and cell proliferation, respectively, by ≥50% compared to the negative controls.

				Toxic Response of Water Extract of Thawed Fruiting Bodies in Test Cell Solution *%*		Toxins According to the Literature	
Ascomycetes	Basidiomycetes		In Vivo *	Ex Vivo BSMI **	In Vitro ICP ** PK-15	Known Toxins	Ref.
**Poisonous−Classified** [19] **Mushrooms (n = 8)**							
	x	*Amanita muscaria*	+	(+) ≤1	(−) 3	Muscimol, ibotenic acid	[12]
	x	*Amanita porphyria*	+	(+) ≤1	(−) 3		
	x	*Amanita virosa*	+	(+) ≤1	(+) 1	Amatoxins	[17]
	x	*Galerina marginata*	+	(−) >1	(+) 0.6	Amatoxins	[21]
x		*Gyromitra esculenta*	+	(+) ≤1	n.a.	Gyromitrin	[18]
	x	*Inocybe geophylla*	+	(+) ≤1	(+) 1	Muscarine	[13]
	x	*Lactarius helvus*	+	(+) ≤1	(+) 0.2	Sesquiterpenes	[19]
	x	*Paxillus involutus*	+	(+) ≤1	(−) 3		[14]
**Non−Edible** [19] **Mushrooms (n = 16)**							
	x	*Calocera viscosa*	(+)	(−) >1	(−) 5		
	x	*Clitocybe fragrans*	n.a.	(+) ≤1	(−) 5		
	x	*Clitocybe vibecinna*	n.a.	(+) ≤1	(−) 5		
	x	*Cortinarius* *armillatus*	(+)	(+) ≤1	(−) 3	Orellanine	[15]
	x	*Cortinarius* *collinitus*	(+)	(−) >1	(−) 5		
	x	*Cortinarius crassus*	(+)	(−) >1	(−) 5		
	x	*Cortinarius* *traganus*	(+)	(−) >1	(−) 5		
	x	*Hygrocybe conica*	(+)	(+) ≤1	(−) >5		
	x	*Hygrophorus* *erubescens*	(+)	(+) ≤1	(−) >5		
x		*Leotia lubrica*	(+)	(−) >1	(+) 0.6		[11]
	x	*Megacollybia platyphylla*	(+)	(+) ≤1	(+) 0.04		
	x	*Phaeolepiota aurea*	(+)	(−) >1	(+) 1		
	x	*Pholiota lenta*	(+)	(−) >1	(+) 1		
	x	*Pholiota squarrosa*	(+)	(−) >1	(+) 1	Squarrosidine Pinillidine	[16]
	x	*Tapinella atrotomentosa*	(+)	(+) ≤1	(+) 0.2		
	x	*Tricholoma equestre*	(+)	(−) >1	(−) >5		
**Edible** [19] **Mushrooms (n = 18)**							
	x	*Albatrellus ovinus*	−	(−) >1	(+) 0.6		
	x	*Agaricus bisporus*	−	(−) >1	(−) 3		
	x	*Agaricus sylvicola*	−	(−) >1	(−) >5		
	x	*Amanita fulva*	−	(−) >1	(−) >5		
	x	*Ampulloclitocybe clavipes*	−	(−) >1	(−) 3		
	x	*Cantharellus* *cibarius*	*−*	(−) >1	(−) *3*		
	x	*Cortinarius* *caperatus*	−	(−) >1	(−) >5		
	x	*Clitocybe odora*	−	(+) ≤1	(−) 3		
	x	*Craterellus* *cornucopioides*	−	(−) >1	(−) 3		
	x	*Craterellus* *tubaeformis*	−	(−) >1	(−) 5		
	x	*Hydnum rufescens*	−	(−) >1	(−) 3		
	x	*Kuehneromyces mutabilis*	−	(−) >1	(−) 3		
	x	*Leccinum scabrum*	−	(−) >1	(+) 1		
	x	*Lentinula edodes*	−	(−) >1	(−) >5		
	x	*Lycoperdon* *perlatum*	−	(−) >1	(−) 5		
x		*Morchella elata*	−	(−) >1	n.a.		
	x	*Phlegmacium triumphans*	−	(−) >1	(−) 3		
	x	*Suillus variegatus*	−	(−) >1	(−) >5		
**Edible after Specific Pretreatments** [19] (n = 4) ***							
	x	*Lactarius rufus*	−	(−) >1	(−) >5		
	x	*Lactarius turpis*	−	(−) >1	(−) 5		
	x	*Lactarius vietus*	−	(−) >1	(−) >5		
	x	*Leccinum versipelle*	−	(+) ≤1	(−) 3		
Negative control		H_2_O		(−) > 2%	(−) > 5%		

* + = poisonous mushroom, (+) = non-edible mushroom, − = edible mushroom.** All tests were performed in triplicate, and the average difference of two-fold dilutions was within one dilution step. *** 2023 species-specific instructions by the Finnish Food Authority.

**Table 3:** Toxicity of colony suspensions of selected *Ascomycota* fungi isolated from water-damaged buildings connected to building-related health complaints. A positive (+) result indicates that 1% and 2.5% of the suspension caused boar sperm motility inhibition (BSMI) and inhibition of cell proliferation (ICP), respectively, by ≥50% compared to the control.

	Isolate Identifier		Toxicity after 2 d of Exposure				
Species	Culture Collection Code	Lab Code	Identified Toxins	BSMI ^2^	ICP ^2^	Origin	Ref.
*Acremonium exuviarum*	DSM 21752 ^1^ FBCC2543 ^2^	BMB4	Acrebol	+	−	Russia	[6]
*Acrostalagmus luteoalbus*	SZMC 26545 ^3^	POB8	Melinacidins	+	+	Finland	[62]
*Aspergillus calidoustus*	SZMC 22623	MH4	Ophiobolins	+	+	Finland	[52]
*Aspergillus flavus*	SZMC 24476	7D/SKK = 7D	Not identified ^5^	−	+	Finland	[24]
*Aspergillus fumigatus*	SZMC 28215	AE1	Not identified ^5^	−	+	Finland	[24]
*Aspergillus niger*	SZMC 27930	Asp21	Not identified ^5^	−	+	Finland	[24]
*Aspergillus versicolor*	FBCC 2549	SL/3	Sterigmatocystin	−	+	Finland	[53]
*Aspergillus westerdijkiae*	FBCC 2553	PP2	Avrainvillamide Stephacidin B Ochratoxin	+	+	Finland	[24] [51]
*Chaetomium globosum*	SZMC 26534	MTAV35	Chaetoglobosin A+C	+	+	Finland	[47]
*Chaetomium cochliodes*	SZMC 24452	OT7	Chaetomin	−	+	Finland	[47]
*Cladosporium* sp.	n.a.	C11	Not identified ^5^	−	+	Finland	This study
*Epicoccum* sp.	FBCC 2565	EMI	Not identified ^5^	−	+	Finland	This study
*Aspergillus pseudoglaucus*	SZMC 27933	8/SL	Not identified ^5^	+	+	Finland	This study
*Penicillium chrysogenum*	SZMC 22627	RUK2/3	Meleagrin	−	+	Finland	[59]
*Penicillium expansum*	SZMC 26543 FBCC2596 CBS 145620 ^4^	RcP61 = P61	Communesin Chaetoglobosins	+	+	Finland	[59]
*Penicillium glabrum*	n.a.	PG21	Not identified ^5^	−	+	Finland	This study
*Paecilomyces variotii*	FBCC 2550	Paec2	Viriditoxin	+	−	Denmark	[24]
*Paecilomyces* sp.	FBCC2628	ST32	Not identified ^5^	−	+	Finland	[24]
*Stachybotrys chartarum*	n.a.	RT	Satratoxin G	−	+	Finland	[4]
*Trichoderma atroviride*	SZMC 12750	H1/226	Trichorzianines	+	+	Finland	[45]
*T. longibrachiatum*	SZMC Thg	Thg	Trilongins	+	+	Finland	[45,54]
Negative control (% *v*/*v*)	Methanol			−(2%)	−(5%)		
				−	−	Finland	

^1^ DSM: DSMZ-German Collection of Microorganisms and Cell Cultures; ^2^ FBCC: Fungi in the Microbial Domain Biological Resource Centre HAMBI, University of Helsinki; ^3^ SZMC: Szeged Microbiology Collection; ^4^ CBS: Westerdijk Institute strain collection; n.a.: not available. All tests were performed in triplicate, and the average difference of two-fold dilutions was within one dilution step. ^5^ Not identified: We have not yet succeeded to identify the toxin of this strain.

**Table 4:** Toxicity endpoints of selected mycotoxins and fungal metabolites (n = 18) produced by indoor mold isolates and xenobiotic chemicals (n = 6). The BSMI assay measured inhibition of boar sperm motility. The in vitro ICP assay measured inhibition of cell proliferation in the continuous cell lines PK-15 (porcine kidney epithelial cells) and/or FFL (feline fetus lung cells). Mitochondrial toxicity was measured as accelerated glucose consumption in differentiated PK-15 cells exposed in monolayer for 48 h. Responses read as toxic are marked as (+), nontoxic as (−), respectively.

		EC_50_ μg mL^–1^			
In Vivo ^3^		Ex Vivo BSMI ^4^	In Vitro ICP ^4^	Mitochondrial Toxicity ^5^	
		1–3d	2d	1d	Ref.
**Mitochondrial Toxins (LD_50_ rat p.o. <1000 mg kg ^–1^ Classified as Toxic in vivo +.)**					
*Acrebol* ^1^	?	(+) 0.1	(−) >10	(+) 0.1	[6]
*Enniatin B* ^2^	+	(+) 0.5	(−) 15	(+) 2	[49]
*Moniliformin* ^2^	+	(+) 2	(−) >20	(+) 2	[50]
*Emodin* ^2^	?	(+) 4	(−) 45	(+) 3	[48]
**Toxins Inhibiting the Glucose Transport**					
*Chaetoglobosin A* ^2^	+	(+) <1	(+) 3	ND	[47]
*Alamethicin* ^2^	+	(+) 0.5	(+) 6	ND	[45]
*Trilongins* ^1^	?	(+) 0.4	(+) 5	ND	[45]
*Trichorzianines* ^1^	?	(+) 0.5	(+) 5	ND	[45]
**Toxins with Undefined Function**					
*Ophiobolin*	+	(+) 0.3	(+) 0.1		[46]
*Avrainvillamide* ^1^	?	(+) 0.2	(+) 0.2	ND	[51]
*Stephacidin B* ^1^	?	(+) 0.3	(++) 0.3	ND	[51]
*Citrinin* ^2^	+	(−) 20	(+) 10		[51]
**Toxins Inhibiting Translation**					
*Sterigmatocystin* ^2^	+	(−)>20	(+) 0.1	ND	[52]
*T –2 toxin* ^2^	+	(−) 0.5	(+) 0.0023		[4]
*Satratoxin G* ^1^	?	(−) 5	(+) 0.0009		[4]
*Verrucarin A* ^2^	+	(−) 5	(+) 0.0006		[4]
*Roridin A* ^2^	+	(−) >1	(+) 0.0007		[4]
**Mycotoxins Used as Positive Controls Calculated as the Mean of 10 Measurements**					
Positive control for ICP assays (PK –15 and FFL cells)					
Citrinin	+	≥20	13 (±1.6)		This study
Positive control for the BSMI assay					
Enniatin B	+	0.6 (±0.14)	18 (±7.7)		This study
**Toxins without Response in the Bioassays Calculated from 20 Measurements**					
*Ochratoxin A* ^2^	+	(−) >50	(−) >50	–	[51]
**Xenobiotic Chemicals, LD_50_ Fish Inhaled <10 μg mL ^–1^ = Toxic in vivo Marked as +.**					
*DDAC*	+	(+) 1	(+) 0.5	(+) 1	[44]
*Genapol X 080*	+	(+) 3	(−) 30	(+) 5	[44]
*Tween 80*	−	(+) 10	(−) 500		[44]
*Triton X –100*	+	(+) 1	(−) 30		[44]
*SDS*	+	(+) 10	(−) 120		[44]
*Triclosan*	+	(+) 1	20 (−)	5	[9]
**Positive control for BSMI and ICP assay (PK –15 and FFL cells), calculated as the mean of 11 measurements**					
*Triclosan*	+	1 (±0.6)	13 (±4.2)		This study
**Negative control % (*v*/*v*)**					
*Methanol*		(−) 1%	(−) 10%		This study
*Ethanol*		(−) 1%	(−) 10%		This study

^1^ Identified and purified from producer strain. ^2^ Commercial source. ^3^ Toxicity to experimental animals according to safety sheets of Sigma-Aldrich. ^4^ All tests were performed in triplicate, and the maximal difference of two-fold dilutions was one dilution step. ^5^ Measured as acceleration of glycolysis and depolarization of mitochondria. ND = Not detected in sublethal concentrations.

**Table 5:** Toxicity of methanol extracts of dusts from moldy houses and dry houses in BSMI, ICP, and mitochondrial toxicity tests with boar sperm and the somatic cells PK-15. In the BSMI and ICP assays, an EC_50_ < 25 μg mL^−1^ and EC_50_ < 50 μg mL^−1^ were classified as toxic, respectively. For mitochondrial toxicity, a positive readout was interpreted if acceleration of glycolysis was observed at <25 μg mL^−1^, and >four-times-lower exposure concentrations than the lethal toxicity.

Toxicity				
Reported Health Complaints In Vivo		Ex Vivo BSMI	In Vitro ICP	Mitochondrial Toxicity ^1^
Wet Buildings Containing Visible Mold Growth (n = 12)				
A	+	(−) >50	(−) >100	−
B	+	(−) >50	(−) >100	−
C	+	(−) >50	(−) >100	−
D	+	(+) <25	(+) <50	(+)
E	+	(−) >50	(−) >100	−
F	+	(+) <25	(−) >100	(+)
G	+	(−) >50	(−) >100	
H	+	(−) >50	(−) >100	−
I	+	(+) <25	(+) <50	(+)
J	+	(+) <25	(−) >100	
K	+	(−) >50	(−) >100	
L	+	(+) <25	(−) >100	
Dry, mold−free buildings (n = 14)				
1	−	(+) <25	(+) <50	(+)
2	−	(+) <25	(+) <50	(+)
3	−	(+) <25	(−) >100	(+)
4	−	(+) <25	(+) <50	(+)
5	−	(+) <25	(−) >100	(+)
6	−	(−) >50	(−) >100	(−)
7	−	(−) >50	(−) >100	(−)
8	−	(+) <25	(+) <50	(+)
9	−	(+) <25	(−) >100	(+)
10	−	(+) <25	(−) >100	(−)
11	−	(−) >50	(−) >100	(−)
12	−	(−) >50	(−) >100	(−)
13	−	(−) >50	(−) >100	(−)
14	−	(−) >50	(−) >100	(−)
Positive controls				
Dusts from spoiled feed and bedding ^3^ (n = 6)	+	(+) <25	(+) <50	(+)
Hay dust spiked with valinomycin 1000 μg g^−1^		(+) <0.1	>5	(+)
Negative controls				
Hay dust (good quality hay) (n = 10) ^2^	−	(−) >50	(−) >100	(−)
Methanol % (*v*/*v*)	+	(−) <1%	(−) <10%	(+)

^1^ Measured as accelerated glycolysis and/or as a decrease in mitochondrial membrane potential ΔΨm < 100 mV. ^2^ No health complaints reported. ^3^ Health complaints reported, visibly contaminated by microbes. All tests were performed in triplicate, and the average difference of two-fold dilutions was within one dilution step.

**Table 6:** Summary of toxic responses in the two complementary bioassays, ICP and BSMI, exhibited by mushrooms (n = 46), Ascomycota mold colonies (n = 21), fungal secondary metabolites (n = 18), xenobiotic chemicals (n = 6), and extracts of indoor settled dusts (n = 23).

Proportion of Positive Responses in Total Positive Test Responses Obtained in the Two Bioassays (%)					
	Total ICP- Positive	Total BSMI-Positive	ICP- + BSMI- Positive	Only ICP- Positive	Only BSMI-Positive
Mushrooms (n = 46)	26	35	11	15	22
Indoor molds (n = 21)	90	48	38	52	10
Fungal metabolites (n = 18)	72	67	44	28	22
Xenobiotic chemicals (n = 6)	33	100	33	none	83
Settled dusts (n = 23)	23	50	26	none	27

## Data Availability

Data supporting the reported results are available upon reasonable request from the corresponding author.

## Supplementary Materials

The following supporting information can be downloaded at: https://www.mdpi.com/article/10.3390/pathogens13030217/s1, Supplementary material, Figure S1: Morphotypes of indoor molds recognized by macroscopic (a) and microscopic inspection (b).

## Appendix A

GenBank accessions of the ITS sequences obtained in this study

**GenBank Accession**	**Species Name**
PP165371	*Amanita fulva*
PP152353	*Amanita virosa*
PP152355	*Galerina marginata*
PP152354	*Megacollybia platyphylla*
PP165373	*Tricholoma* sp.
PP165374	*Albatrellus ovinus*
PP165376	*Tapinella atrotomentosa*
PP165377	*Cortinarius* sp.
PP165372	*Hygrophorus agathosmus*
PP165375	*Clitocybe nebularis*
