# Supplementary material for: Toxicity Screening of Fungal Extracts and Metabolites, Xenobiotic Chemicals, and Indoor Dusts with In Vitro and Ex Vivo Bioassay Methods
Source: Pathogens. 2024 Feb 29;13(3):217. doi: 10.3390/pathogens13030217 (PMC10974995; doi:10.3390/pathogens13030217)
Supplement: Supplementary file 1 [file pathogens-13-00217-s001.zip › pathogens-2868403-supplementary.pdf]

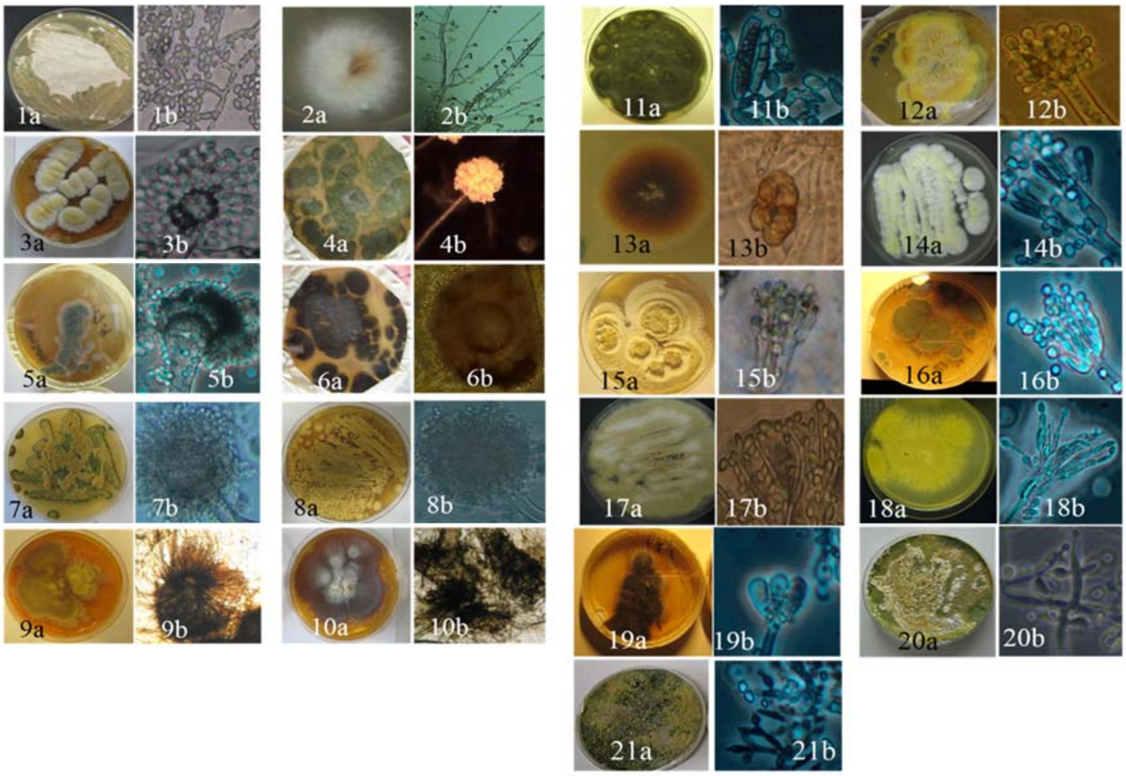

Figure S1. Morphotypes of indoor molds recognized by macroscopic (a) and microscopic inspection (b). The colonies on malt extract agar and the micrographs were pictured after 1- 2 weeks of incubation at room temperature. Panel 1 = *Acremonium exuviarum* strain BMB4, Panel 2 = *Acrostalagmus luteoalbus* strain POB8, Panel 3 = *Aspergillus calidoustus* strain MH4, Panel 4 = *Aspergillus flavus* strain 7 D, Panel 5 = *Aspergillus fumigatus* strain AEI, Panel 6 = *Aspergillus niger* strain Asp 21, Panel 7 = *Aspergillus versicolor* strain SL/3, Panel 8 = *Aspergillus westerdijkiae* strain PP2, Panel 9 = *Chaetomium globosum* strain MTV 35, Panel 10 = *Chaetomium cochliodes* strain OT7, Panel 11 = *Cladosporium* sp. strain C11, Panel 12 = *Aspergillus pseudoglaucus* (former *Eurotium herbariorum*) strain 8/SL, Panel 13 = *Epicoccum* sp. strain EMI, Panel 14 = *Penicillium chrysogenum* strain RUK2/3, Panel 15 = *Penicillium expansum* strain RcP61, Panel 16 = *Penicillium glabrum* strain PG 21, Panel 17 = *Paecilomyces variotii* strain Paec2, Panel 18 = *Paecilomyces* sp. strain ST32, Panel 19 = *Stachybotrys chartarum* strain RT, Panel 20 = *Trichoderma atroviride* strain H1/226, Panel 21 = *Trichoderma longibrachiatum* strain Thg.
